# uniSynK: A Ratio-Optimized Bicistronic Chemically Induced Dimerization Tool for Robust Induction of Dendritic Spine Enlargement

**DOI:** 10.1523/ENEURO.0157-26.2026

**Published:** 2026-09-29

**Authors:** Kota Homma, Masanari Ohtsuka, Siqi Zhou, Hitoshi Okazaki, Tomoki Arima, Haruo Kasai, Takeshi Sawada

**Affiliations:** ^1^Laboratory of Structural Physiology, Center for Disease Biology and Integrative Medicine, Faculty of Medicine, The University of Tokyo, Tokyo 113-0033, Japan; ^2^International Research Center for Neurointelligence (WPI-IRCN), UTIAS, The University of Tokyo, Tokyo 113-0033, Japan; ^3^Department of Physiology, Graduate School of Medicine, The University of Tokyo, Tokyo 113-0033, Japan

**Keywords:** dendritic spine, IRES, Kalirin-7, synaptic plasticity

## Abstract

Synaptic plasticity is widely implicated in learning and brain-state regulation. Although correlations between synaptic modifications and diverse brain functions have been extensively reported, establishing causality requires tools that can directly and selectively perturb synapses in vivo. Recently, the chemogenetic actuator SYNCit-K (SynK) enabled causal induction of dendritic spine enlargement and synaptic potentiation. However, SynK relies on the coexpression of two independent components delivered by separate viral vectors, making it difficult to reliably achieve appropriate expression levels of both components within the same neuron. Here, using neocortical dissociated cultures from mouse embryos of undetermined sex, we sought to develop a unified, single-vector version of SynK (uniSynK) that enables robust and safe induction of dendritic spine enlargement. We first found that each SynK component induces distinct, concentration-dependent abnormalities in neuronal morphology when overexpressed, underscoring the need for precise control of their expression ratio. To address this challenge, we engineered a single construct in which the two components are linked by internal ribosome entry site (IRES) variants with graded translational efficiencies. Systematic screening of IRES variants identified an optimized configuration that preferentially drives each component within its respective concentration range that preserves normal spine morphology. The optimized uniSynK efficiently induces dendritic spine enlargement while exhibiting fewer off-target morphological abnormalities than observed in the multivector system when expression levels were not properly controlled. By simplifying experimental design, this single-vector, ratio-optimized synaptic chemogenetic tool provides a practical and broadly applicable platform that should facilitate causal interrogation of synaptic plasticity across neural circuits and behavior.

## Graphical abstract

**Figure:**
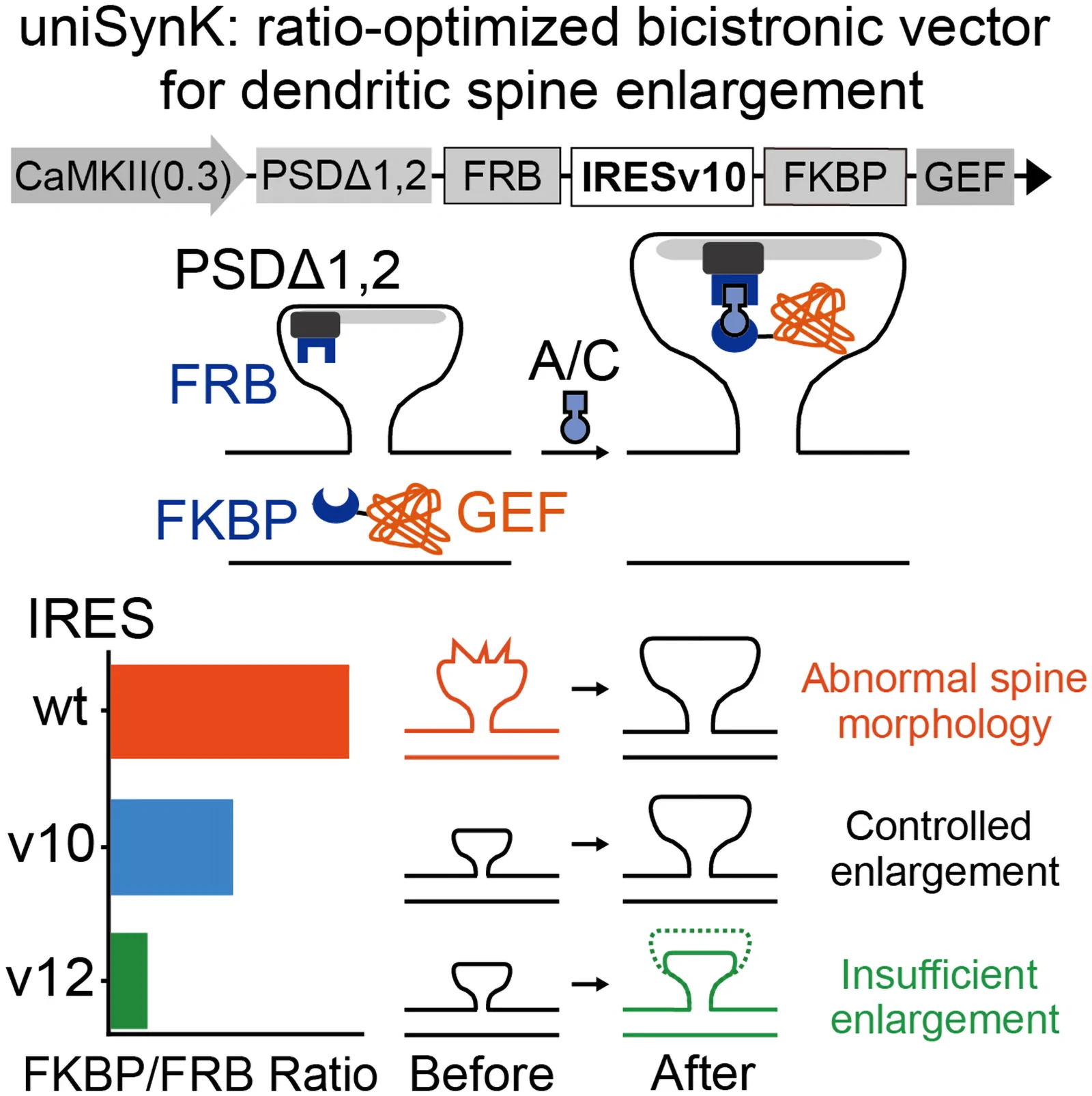
Visual Abstract

## Significance Statement

Synaptic plasticity is thought to shape learning, brain states, and neuropsychiatric disease, but testing its causal roles requires tools that can directly manipulate synapses with minimal side effects. Here, we developed uniSynK, a single-vector chemogenetic system that induces dendritic spine enlargement by coordinating the expression of two functional components. By characterizing the expression level-dependent effects of each component and using IRES variants to optimize their expression ratio, uniSynK achieves robust spine enlargement while reducing unintended morphological abnormalities associated with imbalanced expression. This work provides both a practical tool for synaptic manipulation and a general strategy for optimizing multicomponent biological systems.

## Introduction

Synaptic plasticity is a fundamental mechanism through which the brain adapts to experience. Long-term potentiation (LTP), for example, has been intensively studied as a cellular correlate of learning (Matsuzaki et al., 2004; Xu et al., 2009; Kasai et al., 2021), whereas alterations in dendritic spines—the structural substrates of excitatory synapses—including changes in number, morphology, and associated genes, have been linked to psychiatric conditions (Penzes et al., 2011; McCann and Ross, 2017). Recent studies have further suggested that synaptic plasticity is dynamically regulated across brain states, with prolonged wakefulness leading to dendritic spine enlargement and accumulation of excitatory synaptic receptors (de Vivo et al., 2017; Diering et al., 2017).

Despite these associations, the causal roles of these synaptic changes remain incompletely understood. Addressing this challenge requires tools that enable selective manipulation of defined synapses. Optogenetic approaches have been widely developed over the past two decades, with a subset adapted to manipulate synaptic plasticity (Hayashi-Takagi et al., 2015; Murakoshi et al., 2017; Goto et al., 2021; Shibata et al., 2021). While offering high spatial and temporal resolution, they are less suited for chronic or large-scale manipulation due to limitations such as tissue heating and the need for invasive optical hardware.

To circumvent these limitations, a chemogenetic tool to induce dendritic spine enlargement and correlated synaptic potentiation (Matsuzaki et al., 2004; Holler et al., 2021), SYNCit-K (Synapse-targeted Chemically induced translocation of Kalirin-7; SynK), was recently developed (Sawada et al., 2024). SynK employs rapamycin-dependent FKBP–FRB dimerization (Inoue et al., 2005) to mimic the physiological shuttling of Kalirin-7, a Rac1-activating guanine nucleotide exchange factor (GEF), into postsynaptic densities (PSD; Xie et al., 2007), thereby enlarging dendritic spines and strengthening excitatory synapses. Importantly, SynK enables causal interrogation of synaptic potentiation at the behavioral level, as demonstrated by increased NREM sleep and δ-power following SynK-induced potentiation of prefrontal cortical synapses (Sawada et al., 2024).

Despite these advances, the limited packaging capacity of AAV (<4.7 kb; Halbert et al., 2002; Tornabene et al., 2019) makes it challenging to accommodate the two components—one encoding a truncated form of Kalirin-7 (K7GEF-Cterm) conjugated with FKBP (FKBP–K7GEF–Cterm) and the other a PSD-anchored FRB array (PSDΔ1,2–FRB)—such that the current system relies on two separate AAVs.

AAV-mediated expression typically varies across infected neurons and across the infection site (Tian et al., 2009; Chen et al., 2013). As a result, achieving an appropriate expression level—sufficient to elicit an effect while avoiding overexpression-related side effects (Dana et al., 2014; Moriya, 2015; Mori et al., 2020; Goossens et al., 2021)—in a large proportion of cells remains challenging. This challenge is exacerbated when multiple AAVs are used, as coordinated expression of all components at appropriate levels is required within the same cells, thereby reducing the fraction of cells meeting these conditions and complicating the application of the system (Grieger et al., 2006; Maturana et al., 2023), a problem that may be further compounded by variability across AAV preparations. One strategy to mitigate this limitation is to place both components within a single AAV construct by compacting the design, thereby coupling their expression and facilitating coordinated control of their levels.

In this study, we first attempted to shorten the coding sequences of the two SynK components to enable packaging into AAV vectors, using dissociated neuronal cultures. We then determined the maximum expression levels of each component that did not cause off-target effects in baseline conditions. To further streamline the system, we redesigned SynK as a single-vector construct by linking the two components via an internal ribosome entry site (IRES). By selecting IRES variants with graded translational efficiencies (Koh et al., 2013), we fine-tuned the FKBP/FRB expression ratio, allowing each component to be expressed near its maximal level without detectable off-target effects. Notably, the optimized unified-SynK construct (uniSynK) robustly induced dendritic spine enlargement while exhibiting fewer side effects than the original split-vector design which exhibited variability in the expression levels of the two components. This one-vector, ratio-optimized uniSynK should simplify future in vivo applications and provide a practical platform for causal interrogation of synapse–behavior relationships.

## Materials and Methods

All animal procedures followed the guidelines of the Animal Experimental Committee of the Faculty of Medicine at the University of Tokyo.

### Plasmid and AAV preparation and purification

Plasmids were constructed using standard molecular cloning techniques, including restriction enzyme digestion, PCR amplification, and In-Fusion assembly (Takara Bio). EMCV IRES variants (v10, v12; Koh et al., 2013) were generated by PCR-based mutagenesis. All constructs were sequence-verified and prepared using endotoxin-free plasmid purification kits (Qiagen):

pCAG-mScarlet-C1
pAAV-hSyn-DIO-mScarlet-WPRE-hGHpolyA
pAAV-CaMKII(0.4)-Cre-WPRE-hGHpolyA
pAAV-CaMKII(0.3)-PSDΔ1,2-FRB-WPRE-hGHpolyA
pAAV-CaMKII(0.3)-PSDΔ1,2-FRB-mTq-WPRE-hGHpolyA
pAAV-CaMKII(0.3)-mVenus-FKBP-Kalirin7GEF-Cterm-WPRE-hGHpolyA
pAAV-CaMKII(0.3)-mVenus-FKBP-Kalirin7GEF-WPRE-hGHpolyA
pAAV-CaMKII(0.3)-PSDΔ1,2-FRB-mTq-IRESwt-mVenus-FKBP-Kalirin7GEF-W3SL
pAAV-CaMKII(0.3)-PSDΔ1,2-FRB-mTq-IRESv10-mVenus-FKBP-Kalirin7GEF-W3SL
pAAV-CaMKII(0.3)-PSDΔ1,2-FRB-mTq-IRESv10-mVenus-FKBP-Kalirin7GEF(K1393A)-W3SL
pAAV-CaMKII(0.3)-PSDΔ1,2-FRB-mTq-IRESv12-mVenus-FKBP-Kalirin7GEF-W3SL
pAAV-CaMKII(0.3)-PSDΔ1,2-FRB-IRESv10-FKBP-Kalirin7GEF-W3SL
pAAV-hSyn-SEP-GluA2-WPRE-hGHpolyA

AAVs were produced, and their titers were measured as described previously (Grieger et al., 2006). In brief, plasmids for the AAV vector, pHelper (Stratagene) and pUCmini-iCAP30 PHP.eB (#103005, Addgene), were transfected into HEK293 cells (AAV293, Stratagene). After 3 d, cells were harvested, and AAVs were purified twice using iodixanol. The titers of AAVs were estimated using quantitative PCR. uniSynK plasmid has been deposited at Addgene.

### Dissociated culture of the neocortex

Dissociated cortical neuron cultures were prepared from embryonic day 17 (E17) ICR mouse embryos (SLC) of both sexes (Okabe et al., 1999). Cells were plated at a density of 4.0 × 10^4^ cells/cm^2^ on poly-l-lysine-coated glass-bottom dishes (Greiner, Advanced TC). Cultures were maintained at 37°C in a humidified atmosphere with 5% CO_2_ in Neurobasal medium supplemented with B27 (1:50; Invitrogen) and l-glutamine (1:200; Invitrogen). Half of the medium was replaced every ∼5 d.

### Plasmid lipofection and AAV transduction to dissociated cortical neurons

For plasmid delivery via lipofection, plasmid mixtures were combined with Lipofectamine 2000 (Invitrogen) and incubated for 20 min. Neurons were transfected at DIV16, and the medium was replaced after 4 h with a 1:1 mixture of precollected conditioned medium and fresh culture medium. For AAV transduction, viral mixtures were added to neurons at DIV11–12, and the medium was replaced with a 1:1 mixture of conditioned medium and fresh culture medium, after 20–24 h.

### Dendritic spine imaging in dissociated cultures and image processing

Time-lapse imaging of up to three fluorescent channels (mTurquoise2, mVenus, and mScarlet) was performed every 20 min using a confocal microscope (A1R, Nikon), equipped with a 60× oil-immersion objective lens (NA 1.40) at DIV18–19. *Z*-stack images were acquired with a step size of 0.3 µm along the *z*-axis, typically spanning ∼30 optical sections to fully encompass the analyzed spines. To activate SynK via induced dimerization, half of the culture medium was removed and retained in advance. Immediately before application, a portion of the remaining medium was mixed with the A/C heterodimerizer (Takara Bio; 0.5 mM stock in ethanol) and then returned to the dish to achieve a final concentration of 4 µM. After 10 min, cells were returned to the retained conditioned medium.

Representative images were rotated when necessary using bilinear interpolation in ImageJ. Spines with more than two spinules were classified as highly branched (Penzes et al., 2001), and their fraction was calculated per dendrite (Fig. 4*F*).

### Measurement of time series changes in spine volume

Time-lapse *z*-projected images were aligned using the ITK registration framework in Python. Signal leakage across channels was quantified using neurons expressing only mScarlet, mVenus, or mTurquoise2 (mTq), and these values were used for spectral unmixing. After processing, the background-subtracted mScarlet fluorescence intensity of each spine head, normalized to the total fluorescence of the imaging area, was used as a proxy for spine-head volume, as described previously (Matsuzaki et al., 2004). Spines (4–20 per dendrite) were randomly selected for analysis. Spines that could not be consistently enclosed within the same ROI across all time points were excluded. In addition, spines lacking detectable mTq signal were excluded from mVenus/mTq ratio analysis. Baseline spine volume was defined as the mean across all time points prior to A/C heterodimerizer application. For quantification and statistical analyses, relative increases in spine volume from 20 to 120 min after A/C heterodimerizer administration were averaged.

In experiments with nonsparse AAV-mediated expression of mVenus and mTq (Fig. 8), to minimize signal contamination from neighboring neurons, a mask was generated by applying 2D Otsu thresholding to the cell-fill mScarlet channel. Regions lacking signal were excluded from the other channels (mTq and mVenus) prior to alignment of *z*-projected images, followed by all subsequent processing described above.

### Measurement of absolute dendritic spine volume and its intrinsic fluctuations

To estimate the absolute spine volume, *z*-stack images were deconvolved in Python using flowdec with the Richardson–Lucy method based on a synthetic point spread function calculated from imaging parameters and then binarized using Otsu's method. The absolute spine volume was calculated by counting the voxels within the binarized spine regions and multiplying the voxel count by the voxel volume. For the analysis of intrinsic spine fluctuations (Figs. 3*B*–*D*, 6*K*,*L*), the mean change in spine volume (μ) and the standard deviation (σ) were calculated over 20 min intervals at 45–25 and 25–5 min before and 35–55 min after A/C heterodimerizer administration, as previously described (Ishii et al., 2018).

### Sholl analysis

Confocal *z*-stack images were imported into Fiji (ImageJ), and dendritic arbors were semiautomatically traced using the SNT plugin (Sholl, 1953; Arshadi et al., 2021). Concentric circles were centered on the soma, and the number of dendritic intersections with each circle was quantified.

### Statistical analysis

Sample sizes were determined empirically based on previous studies and are comparable to those generally used in the field. Data are presented as individual data points with mean ± SEM, where applicable. Unless otherwise specified, statistical analyses were performed using nonparametric methods, with Bonferroni correction applied where appropriate for multiple comparisons. *P* values are reported as unadjusted values, and statistical significance was assessed using corrected significance thresholds.

## Results

### Kalirin-7 C-terminal truncation and fluorescent protein addition do not impair SynK function

To rationally reduce the size of the SynK constructs while preserving its functional core, we revisited the molecular requirements underlying Kalirin-7–mediated spine enlargement. The original SynK contained the DH-PH (GEF module) domain and the C-terminal region containing the PDZ ligand of Kalirin-7 (Fig. 1*A*–*C*; Sawada et al., 2024). While the GEF module is directly responsible for Rac1 activation (Xie et al., 2007), the primary role of the PDZ ligand has been proposed to be subcellular targeting (Lee and Zheng, 2010). Thus, we hypothesized that the PDZ ligand is dispensable in the SynK system, in which rapid and stable recruitment of Kalirin-7 to dendritic spines is artificially achieved via the rapamycin analog (A/C heterodimerizer). To directly test this hypothesis, we removed the C-terminal region from the original construct (Fig. 1*D*). As a result, the truncated SynK construct expressed in dissociated cortical neurons (PSDΔ1,2–FRB, 3 µg/ml; mVenus–FKBP–K7GEF, 0.05 µg/ml) showed K7GEF translocation and spine enlargement (Fig. 1*E*,*F*) that were indistinguishable from those induced by the original construct (Fig. 1*H*), thereby defining the simplified functional unit required for SynK-mediated spine enlargement. All subsequent development was based on this truncated construct.

**Figure 1.**
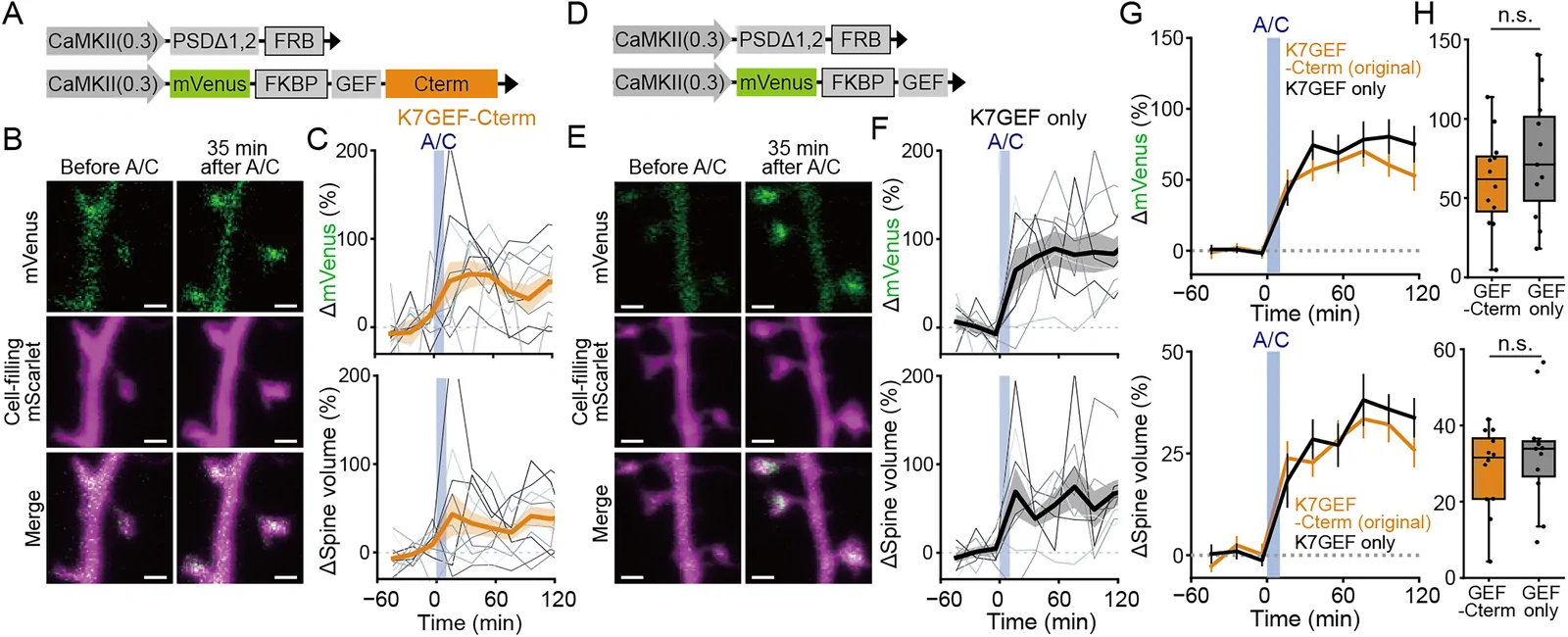
C-terminal truncation of Kalirin-7 preserves spine enlargement in SynK. ***A***, Schematic of the original SynK construct. ***B***, Representative confocal image of a dendrite expressing PSDΔ1,2–FRB (3 µg/ml) and mVenus–FKBP fused to Kalirin-7 GEF and C-terminal regions (0.05 µg/ml) before (−5 min) and after (35 min) application of A/C heterodimerizer. mScarlet signal is shown as a volume marker. Scale bar, 1 µm. ***C***, Time courses of changes in the mVenus signal in dendritic spines (top) and spine volume (bottom), including their averages (bold line) and SEM (shade) for a representative neuron expressing PSDΔ1,2–FRB and mVenus–FKBP–K7GEF–Cterm. Values are plotted as a percentage change from the baseline average. ***D***, Plasmid construct of SynK lacking the Kalirin-7 C-terminal region. ***E***, Representative confocal image of a dendrite expressing PSDΔ1,2–FRB (3 µg/ml) and mVenus–FKBP–K7GEF (without Cterm, 0.05 µg/ml). Scale bar, 1 µm. ***F***, Time course of mVenus and mScarlet signals in dendritic spines for a representative neuron expressing PSDΔ1,2–FRB and mVenus–FKBP–K7GEF. ***G***, ***H***, Averaged time courses (***G***) and summary (***H***) of changes in mVenus signal in dendritic spines (top) and spine volume (bottom) from neurons expressing SynK with either K7GEF–C-terminal region (original) or K7GEF alone. Data in ***G*** are shown as the mean ± SEM. Values in ***H*** represent the average between 35 and 115 min after A/C heterodimerizer administration. *N* (dendrites/neurons) = 12/7 (K7GEF-Cterm) and 11/4 (K7GEF only). The median (horizontal line), quartiles (boxes), and range within 1.5 times the interquartile range (whiskers) are denoted. Statistical comparisons were made using the Mann–Whitney test (top, *U* = 53, *p* = 0.44; bottom, *U* = 58, *p* = 0.64). *p < 0.05; **p < 0.01; n.s., not significant.

To quantitatively assess how the expression levels of the two components influence efficacy and potential side effects, we next modified the system so that both components were fluorescently labeled, fusing mTq to PSDΔ1,2–FRB, while the FKBP–K7GEF component retained its original mVenus tag (Fig. 2*A*). In neurons expressing PSDΔ1,2–FRB–mTq, the mTq signal was localized to dendritic spines (Fig. 2*B*). Following A/C heterodimerizer administration, FKBP–mVenus robustly translocated to dendritic spines, and spine enlargement was comparable to that observed with the original SynK constructs (Fig. 2*B*–*E*). These results indicate that mTq tagging does not interfere with PSDΔ1,2–FRB localization and preserves the functionality of the SynK system.

**Figure 2.**
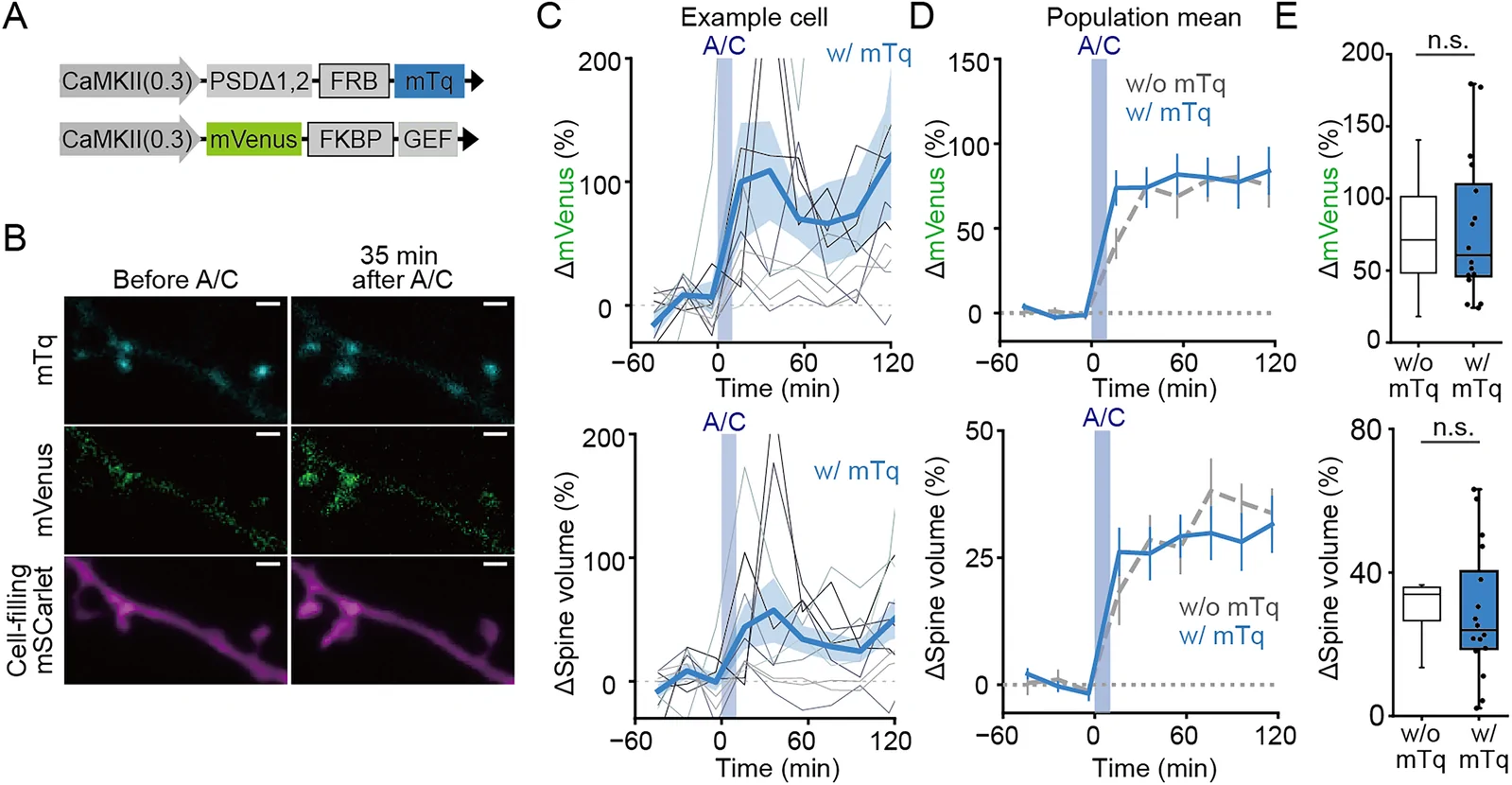
mTq tagging of PSDΔ1,2–FRB does not interfere with SynK function. ***A***, Schematic of the SynK construct with mTq fused to PSDΔ1,2–FRB and lacking the Kalirin-7 C-terminal region. ***B***, Representative confocal images of a dendrite expressing PSDΔ1,2–FRB–mTq and mVenus–FKBP–K7GEF before (−5 min) and after (35 min) application of A/C heterodimerizer. Images of mScarlet signal are shown as a volume marker. Scale bar, 1 µm. ***C***, Time courses of changes in the mVenus signal in dendritic spines (top) and spine volume (bottom), including their averages (bold line) and SEM (shade) for a representative neuron expressing PSDΔ1,2–FRB–mTq and mVenus–FKBP–K7GEF. Values are plotted as a percentage change from the baseline average. ***D***, ***E***, Averaged time courses (***D***) and summary (***E***) of changes in mVenus signal in dendritic spines (top) and spine volume (bottom) from neurons expressing SynK with mTq tagging of PSDΔ1,2–FRB. Data without mTq are reproduced from Figure 1 and shown as dashed lines (***D***) and hollow box plots (***E***) for comparison. Data in ***D*** are shown as the mean ± SEM. Values in ***E*** represent the average between 35 and 115 min after A/C heterodimerizer administration. *N* (dendrites/neurons) = 16/7 (w/ mTq) and 11/4 (w/o mTq). The median (horizontal line), quartiles (boxes), and range within 1.5 times the interquartile range (whiskers) are denoted. Statistical comparisons were made using the Mann–Whitney test (top, *U* = 91, *p* = 0.90; bottom, *U* = 106, *p* = 0.39). *p < 0.05; **p < 0.01; n.s., not significant.

Compared with the original construct (Sawada et al., 2024), removal of the C-terminal region and addition of mTq did not result in more pronounced spine hypertrophy prior to A/C administration (Fig. 3*A*), a phenotype typically observed upon Kalirin-7 overexpression (Xie et al., 2007). In addition, no significant changes in spine fluctuation (Yasumatsu et al., 2008; Minerbi et al., 2009; Ishii et al., 2018) were detected either before or after A/C administration in neurons expressing PSDΔ1,2–FRB–mTq and mVenus–FKBP–K7GEF (Fig. 3*B*–*D*).

**Figure 3.**
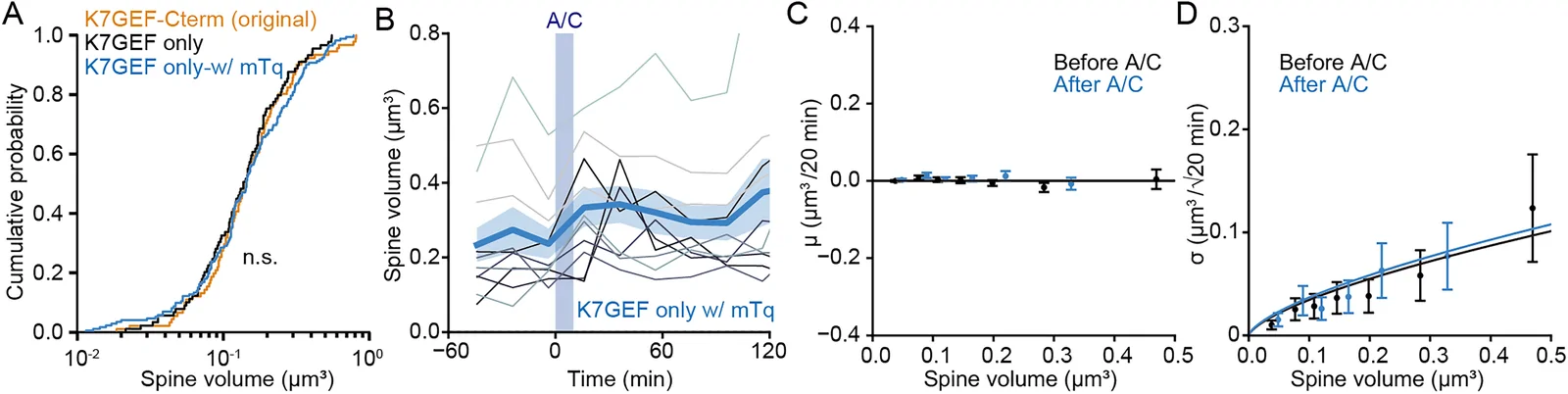
Removal of the Kalirin-7 C-terminal region and addition of mTq does not induce significant changes in spine volume distribution or intrinsic dynamics. ***A***, Cumulative distribution of dendritic spine volumes under three conditions. K7GEF-Cterm (original), *N* = 92; K7GEF only, *N* = 89; K7GEF only-w/ mTq, *N* = 172 spines. Statistical comparisons were made using the Kolmogorov–Smirnov (K–S) test with Bonferroni’s correction. K7GEF-Cterm versus K7GEF only, *D* = 0.065, *p* = 0.97; K7GEF-Cterm versus K7GEF only-w/ mTq, *D* = 0.085, *p* = 0.74. ***B***, Time courses of the estimated absolute volume (see Materials and Methods) of spines before and after. A/C heterodimerizer application, along with their averages (blue line) and SEM (blue shade) for a representative neuron expressing PSDΔ1,2–FRB–mTq and mVenus–FKBP–K7GEF. ***C, D***, Intrinsic spine fluctuation of neurons expressing PSDΔ1,2–FRB–mTq and mVenus–FKBP–K7GEF, before and after A/C heterodimerizer administration, each calculated per 20 min interval. Each plotted point represents the mean (***C***) and the SD (***D***) of spine-head volume changes in 24 pooled spines with similar baseline volumes. Error bars represent SEM values (***C***) and the 95% confidence intervals of the estimated SD (***D***). Data are fitted by a zero (***C***) and to the 2/3 power of the baseline spine volume (***D***). *p < 0.05; **p < 0.01; n.s., not significant.

Collectively, these data indicate that neither truncation of the C-terminal region of Kalirin-7 nor the addition of fluorescent proteins significantly compromises SynK-mediated spine enlargement or introduces detectable morphological abnormalities compared with the original construct.

### The two SynK components each induce concentration-dependent unintended effects on spine morphology under baseline conditions

In developing a single-vector system expressing both components, we first sought to determine the expression range of each component that produced no detectable unintended effects under baseline conditions. Primary cortical neurons were transfected with either mVenus–FKBP–K7GEF (Fig. 4) or PSDΔ1,2–FRB–mTq (Fig. 5). To assess potential side effects across a broad range of expression levels and independently of method-specific influences, we employed both DNA lipofection and AAV-mediated gene delivery.

**Figure 4.**
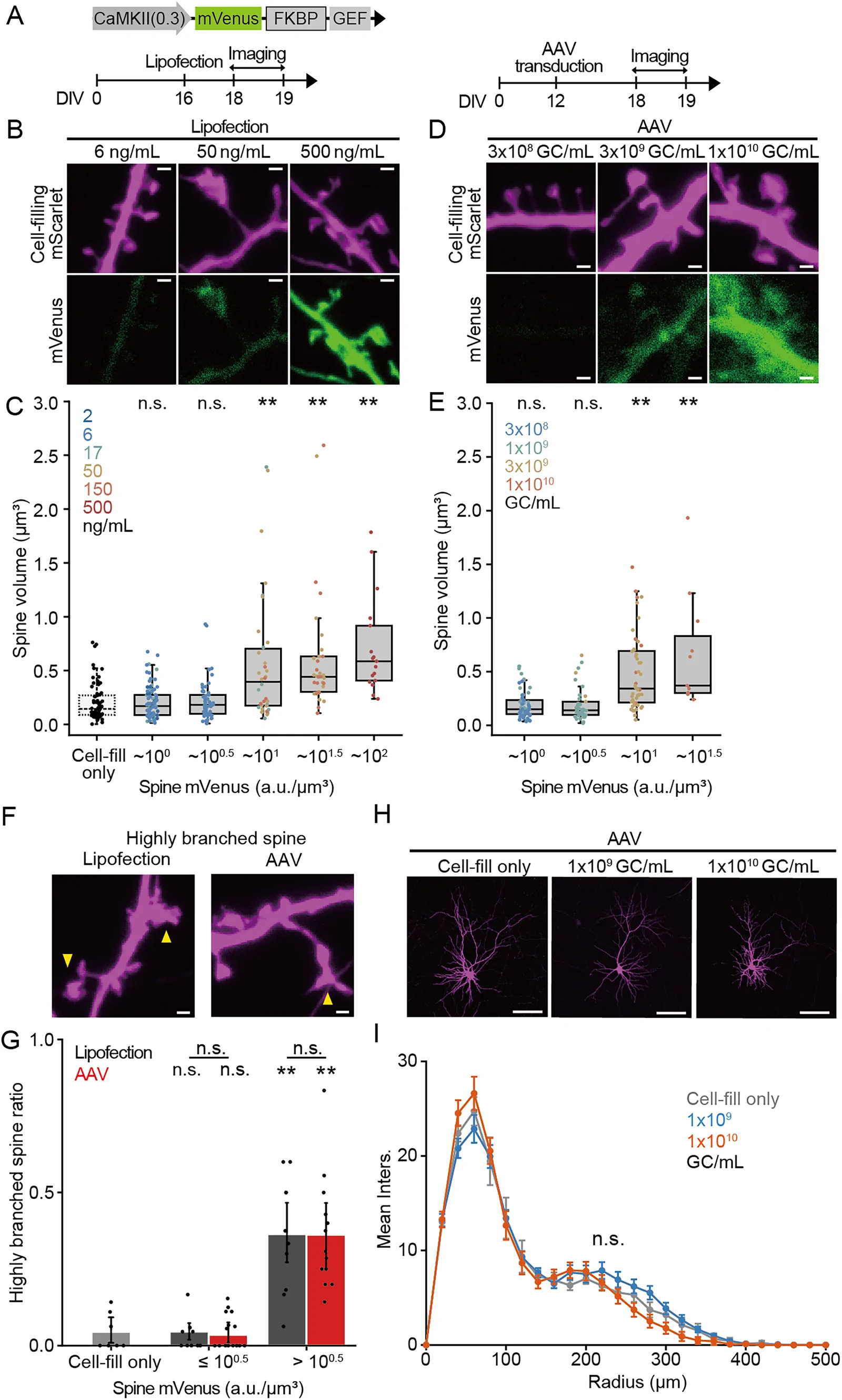
The Kalirin-7 GEF domain induces a concentration-dependent increase in baseline spine volume. ***A,*** Construct and experimental schedule. Two delivery methods were used: lipofection (***B, C***) and AAV (***D, E***). ***B,*** Example confocal images of dendrites expressing mVenus–FKBP–K7GEF via plasmid lipofection. mScarlet signal is shown as a volume marker. Scale bars, 1 µm. ***C,*** Distribution of dendritic spine volumes under plasmid lipofection conditions, plotted as a function of binned spine mVenus intensity. Spine volume was estimated by deconvolution (see Materials and Methods). mVenus intensity was measured for each spine, normalized by the estimated spine volume, and averaged across spines within each dendrite. The mean mVenus intensity per unit volume at 50 ng/ml lipofection was defined as 10 a.u./µm^3^. Six lipofection concentrations (2–500 ng/ml) are shown in different colors. No A/C heterodimerizer was applied. Kruskal–Wallis test (*H* = 81, *p* = 5.5 × 10^−16^) followed by Mann–Whitney test against neurons expressing cell-filling mScarlet alone [*N* (spines/dendrites) = 69/6], with Bonferroni’s correction (−10^0^ a.u./µm^3^, *N* = 85/10, *U* = 2,853, *p* = 0.77; 10^0^–10^0.5^ a.u./µm^3^, *N* = 53/5, *U* = 1,698, *p* = 0.50; 10^0.5^–10^1^ a.u./µm^3^, *N* = 34/5, *U* = 588, *p* = 4.1 × 10^−5^; 10^1^–10^1.5^ a.u./µm^3^, *N* = 33/5, *U* = 310, *p* = 3.1 × 10^−9^; 10^1.5^ a.u./µm^3^, *N* = 17/3, *U* = 96, *p* = 1.0 × 10^−7^). ***D,*** Example confocal images of dendrites expressing mVenus–FKBP–K7GEF via AAV transduction. Sparsely expressed mScarlet, by using a double-floxed inverted open reading frame (DIO) system in conjunction with low Cre expression, is shown as a volume marker. Scale bars, 1 µm. ***E***, Distribution of dendritic spine volumes under AAV transduction conditions, plotted as a function of binned spine mVenus intensity as in ***C***. Four different AAV concentrations (3.0 × 10^8^ –1.0 × 10^10^ GC/ml) are shown in different colors. Kruskal–Wallis test (*H* = 55, *p* = 2.7 × 10^−11^) followed by Mann–Whitney test against neurons expressing cell-filling mScarlet alone with Bonferroni’s correction [−10^0^ a.u./µm^3^, *N* (spines/dendrites) = 60/5, *U* = 1,946, *p* = 0.56; 10^0^–10^0.5^ a.u./µm^3^, *N* = 37/5, *U* = 1,256, *p* = 0.89; 10^0.5^–10^1^ a.u./µm^3^, *N* = 52/7, *U* = 751, *p* = 4.8 × 10^−8^; 10^1^–10^1.5^ a.u./µm^3^, *N* = 11/2, *U* = 95, *p* = 7.2 × 10^−5^]. ***F***, Representative images of highly branched dendritic spine (yellow arrow heads). Scale bars, 1 µm. ***G***, Fraction of highly branched spines per dendrite expressing mVenus–FKBP–K7GEF via lipofection (gray) or AAV (red). Statistical analysis was performed using the Mann–Whitney test with Bonferroni’s correction. Lipofection −10^0.5^ a.u./µm^3^ [(*N* (dendrites) = 15] versus Cell-fill only (*N* = 7), *U* = 51, *p* = 0.91; AAV −10^0.5^ a.u./µm^3^ (*N* = 10) versus Cell-fill only, *U* = 34, *p* = 0.82; Lipofection 10^0.5^- a.u./µm^3^ (*N* = 13) versus Cell-fill only, *U* = 0.0, *p* = 3.4 × 10^−4^; AAV 10^0.5^- a.u./µm^3^ (*N* = 9) versus Cell-fill only, *U* = 2.5, *p* = 1.6 × 10^−3^; −10^0.5^ a.u./µm^3^ lipofection versus AAV, *U* = 60, *p* = 0.65; 10^0.5^- a.u./µm^3^ lipofection versus AAV, *U* = 68, *p* = 0.90. ***H***, Representative images of AAV-transduced neurons expressing mVenus–FKBP–K7GEF. Scale bars, 100 µm. ***I***, Sholl analysis showing the number of dendrite intersections as a function of the distance from the soma. Data are presented as mean ± SEM. Two-way mixed ANOVA: Group × radius interaction, *F* = 1.1, *p* = 0.31; *N* = 12 (cell-fill only), 15 (mVenus–FKBP–K7GEF, 1 × 10^9^ GC/ml), 14 (mVenus–FKBP–K7GEF, 1 × 10^10^ GC/ml). *p < 0.05; **p < 0.01; n.s., not significant.

**Figure 5.**
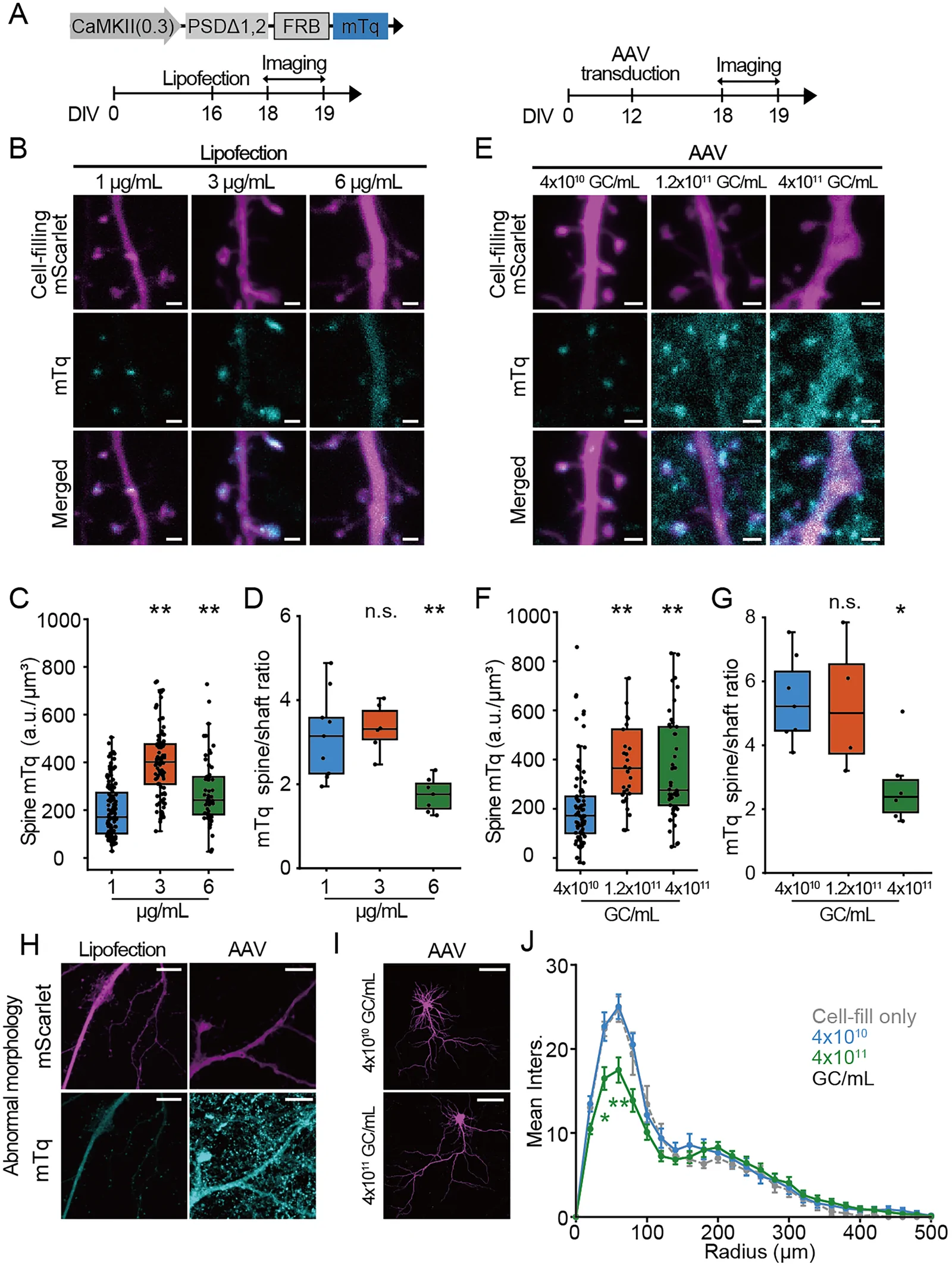
PSDΔ1,2–FRB–mTq induces concentration-dependent spillover into the dendritic shaft and morphological abnormalities. ***A***, Construct and experimental schedule. Two delivery methods were used: lipofection (***B–D***) and AAV (***E–G***). ***B***, Example confocal images of dendrites expressing PSDΔ1,2–FRB–mTq via plasmid lipofection. mScarlet signal is shown as a volume marker. Scale bars, 1 µm. ***C,*** Distribution of mTq signal intensity in dendritic spines across lipofection concentrations (1–6 µg/ml). mTq intensity was normalized to the estimated volume of each spine. Each point represents an individual dendritic spine. *N* (spines/dendrites/neurons) = 111/9/3 (1 µg/ml), 90/6/2 (3 µg/ml), 56/7/5 (6 µg/ml). Statistical comparisons were performed using the Kruskal–Wallis test (*H* = 86, *p* = 2.6 × 10^−19^) followed by the Mann–Whitney test with Bonferroni’s correction (1 versus 3 µg/ml, *U* = 1,339, *p* = 4.9 × 10^−19^; 1 versus 6 µg/ml, *U* = 2,001, *p* = 1.8 × 10^−4^). ***D***, Ratio of mTq signal intensity between dendritic spines and shafts within the same dendrite, across lipofection concentrations (1–6 µg/ml). Signals were normalized by their estimated volumes. Each point represents an individual dendrite. Statistical comparisons were performed using the Kruskal–Wallis test (*H* = 12, *p* = 2.6 × 10^−3^) followed by the Mann–Whitney test with Bonferroni’s correction (1 versus 3 µg/ml, *U* = 23, *p* = 0.69; 1 versus 6 µg/ml, *U* = 59, *p* = 2.1 × 10^−3^). ***E***, Example confocal images of dendrites expressing PSDΔ1,2–FRB–mTq via AAV transduction. Sparsely expressed mScarlet, by using a double-floxed inverted open reading frame (DIO) system in conjunction with low Cre expression, is shown as a volume marker. Scale bars, 1 µm. ***F***, Distribution of mTq signal intensity in dendritic spines across AAV concentrations (4.0 × 10^10^ –4.0 × 10^11^ GC/ml), as in ***C***. *N* (spines/dendrites/neurons) = 83/7/7 (4.0 × 10^10^ GC/ml), 33/4/4 (1.2 × 10^11^ GC/ml), 53/6/6 (4.0 × 10^11^ GC/ml). Kruskal–Wallis test (*H* = 38, *p* = 5.2 × 10^−9^) followed by Mann–Whitney test with Bonferroni’s correction (4.0 × 10^10^ versus 1.2 × 10^11^ GC/ml, *U* = 515, *p* = 1.7 × 10^−7^; 4.0 × 10^10^ versus 4.0 × 10^11^ GC/ml, *U* = 1,125, *p* = 1.6 × 10^−6^). ***G***, Ratio of mTq signal intensity between dendritic spines and shafts within the same dendrite, across AAV concentrations, as in ***D***. The Kruskal–Wallis test (*H* = 7.9, *p* = 0.019) followed by the Mann–Whitney test with Bonferroni’s correction (4.0 × 10^10^ versus 1.2 × 10^11^ GC/ml, *U* = 15, *p* = 0.92; 4.0 × 10^10^ versus 4.0 × 10^11^ GC/ml, *U* = 39, *p* = 8.2 × 10^−3^). ***H***, Representative confocal images of neuronal morphology in neurons transfected with PSDΔ1,2–FRB–mTq by lipofection (6 µg/ml) and infected with AAV-PSDΔ1,2–FRB–mTq (4 × 10^11^ GC/ml). Scale bars, 10 µm. ***I***, Representative image of AAV-transduced neurons expressing PSDΔ1,2–FRB–mTq. Scale bar, 100 µm. ***J***, Sholl analysis showing the number of dendritic intersections as a function of distance from the soma. Data are presented as mean ± SEM. Data for cell-fill only were reproduced from Figure 4*I* for comparison. Two-way mixed ANOVA: Group × radius interaction, *F* = 3.2, *p* = 5.5 × 10^−12^; Tukey's multiple-comparisons test, cell-fill only versus 4 × 10^11^ GC/ml: 40 µm, *p* = 0.018; 60 µm, *p* = 0.0065. *N* = 12, 14, and 16 for cell-fill only, 4 × 10^10^ GC/ml, and 4 × 10^11^ GC/ml, respectively. *p < 0.05; **p < 0.01; n.s., not significant.

Using DNA lipofection at DIV16, we varied the amount of mVenus–FKBP–K7GEF plasmid across six concentrations (2–500 ng/ml), with analyses performed at DIV19 (Fig. 4*A*, left). The mean mVenus signal intensity per spine volume (see Materials and Methods) at 50 ng/ml lipofection was defined as 10 a.u./µm^3^. When the expression exceeded 10^0.5^ a.u./µm^3^, the distribution of spine volumes shifted significantly upward even without A/C heterodimerizer, whereas no significant shift was observed below 10^0.5^ a.u./µm^3^ (Fig. 4*B*,*C*). In AAV-based experiments with transduction at DIV11 (Fig. 4*A*, right), spine volume distributions were likewise shifted at DIV19 only when mVenus–FKBP–K7GEF expression exceeded 10^0.5^ a.u./µm^3^ (Fig. 4*D*,*E*). The comparable expression ranges without unintended effects on spine volume between AAV and lipofection suggest that these effects are primarily determined by the expression level rather than by expression duration or delivery method. An increase in highly branched spines, previously reported with Kalirin-7 overexpression (Penzes et al., 2003; Ma et al., 2008), was also observed in both DNA lipofection and AAV, but only when mVenus–FKBP–K7GEF expression exceeded 10^0.5^ a.u./µm^3^ (Fig. 4*F*,*G*). Only at these high expression levels, Sholl analysis showed a nonsignificant tendency toward increased proximal and decreased distal branching, resembling the pattern previously reported following Kalirin-7 overexpression (Ma et al., 2008; Fig. 4*H*,*I*). From these results, we considered 10^0.5^ a.u./µm^3^ as the maximum expression level of mVenus–FKBP–K7GEF without detectable effects on neuronal morphology.

Similarly, PSDΔ1,2–FRB–mTq was expressed by DNA lipofection at three plasmid concentrations (1–6 µg/ml; Fig. 5*A*, left). Increasing the concentration from 1 to 3 µg/ml significantly elevated PSDΔ1,2–FRB–mTq levels in dendritic spines (the mean spine mTq concentration at 1 µg/ml in lipofection was defined as 200 a.u./µm^3^) without altering the shaft-to-spine expression ratio (Fig. 5*B*–*D*). In contrast, at 6 µg/ml, mTq signal per spine volume did not increase, whereas the spine-to-shaft ratio decreased, suggesting spillover of PSDΔ1,2–FRB from spines to dendritic shafts (Fig. 5*B*–*D*). Consistent with this, in AAV-transduced neurons (Fig. 5*A*, right), spine mTq plateaued at ∼400 a.u./µm^3^ on average, despite increasing expression levels, while the spine-to-shaft ratio decreased, consistent with spillover (Fig. 5*E*–*G*). Because SynK induces spine enlargement by recruiting FKBP–K7GEF to PSDΔ1,2–FRB localized in dendritic spines, excessive expression of PSDΔ1,2–FRB in shafts is likely to compromise the efficacy of the tool. Moreover, at expression levels that induced spillover, neurons frequently exhibited abnormal morphologies, including highly branched spines and lamellipodia-like structures (Fig. 5*H*), an aberrant increase in small spines and filopodia, and significantly decreased dendritic arbor complexity (Fig. 5*I*,*J*), suggesting that mislocalization of PSDΔ1,2–FRB adversely affects cellular integrity. Accordingly, we defined 3 µg/ml (lipofection) and 1.2 × 10^11^ GC/ml (AAV) as the upper limits of the expression range without detectable adverse effects for PSDΔ1,2–FRB–mTq.

### IRESv10-linked SynK induces spine enlargement across a broad expression range without structural abnormalities

To maximize the efficacy of SynK-mediated spine enlargement, each component should be expressed at sufficiently high levels while remaining below the threshold for unintended effects. Given the markedly different thresholds for off-target morphological effects between the two components, we designed a single-construct SynK by linking them with IRES variants (IRESv; Koh et al., 2013) that allow tuning of their relative expression levels (Fig. 6*A*–*C*). Consistent with previous reports, PSDΔ1,2–FRB–mTq expression levels did not differ significantly across IRES variants (Fig. 6*D*), whereas the mVenus–FKBP–K7GEF to PSDΔ1,2–FRB–mTq expression ratio in dendritic spines, normalized to IRESwt, was 0.32 ± 0.03 for IRESv10 and 0.05 ± 0.03 for IRESv12 (Fig. 6*E*). This ratio did not differ significantly when the same IRES variant was used at different plasmid concentrations (1 or 3 µg/ml lipofection), indicating that relative expression levels are robustly determined by each IRES variant (Fig. 6*E*). Together, these results demonstrate that IRES variants enable stable and predictable control of relative protein expression in neurons, consistent with observations in other cell types.

**Figure 6.**
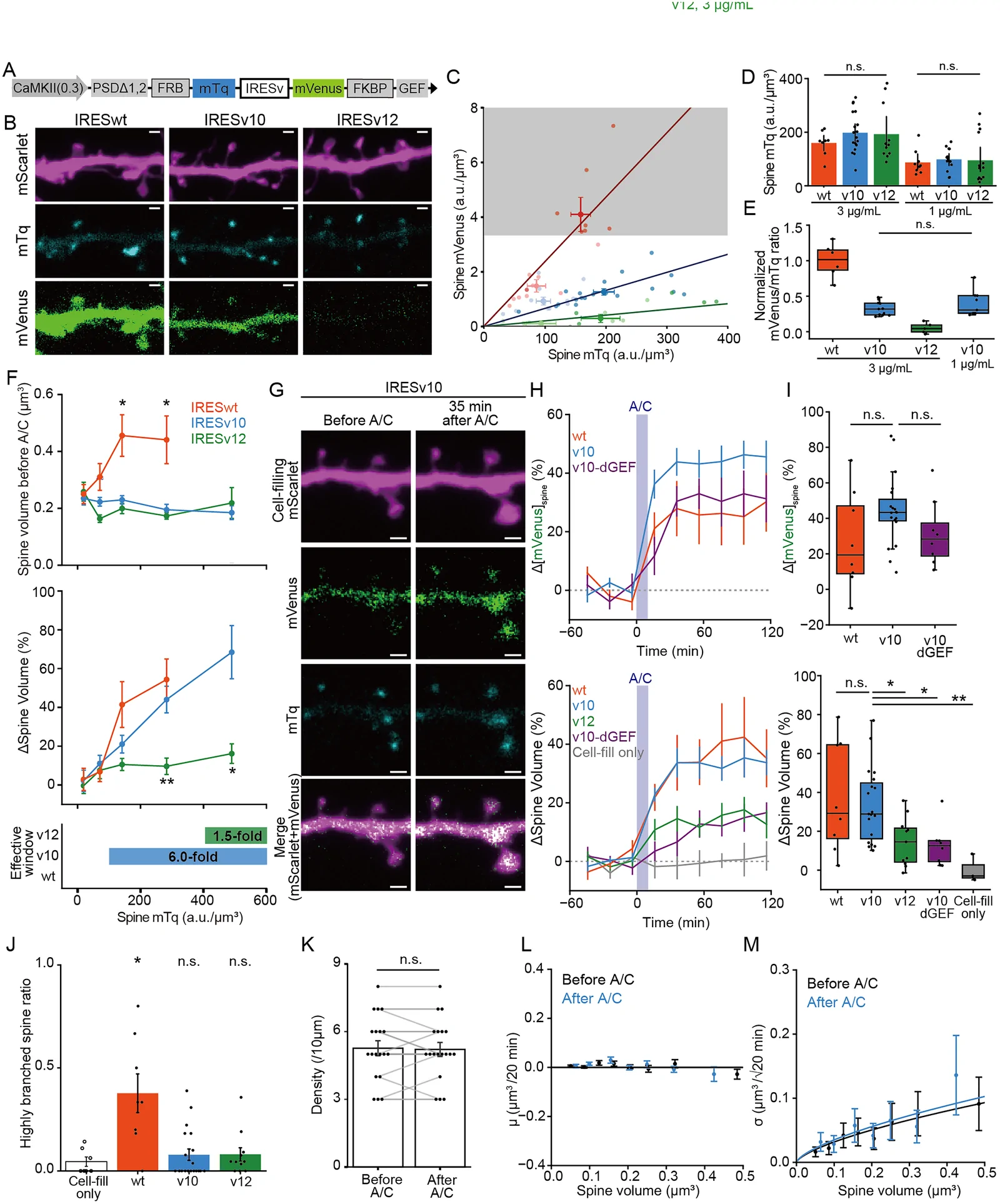
Combining two SynK components using IRESv10 induces dendritic spine enlargement across a broad expression range without detectable structural abnormalities under plasmid lipofection. ***A***, Schematic of a single-vector construct combining two SynK components via IRES variants (IRESv). Three variants with different translation efficiencies were used: IRESwt, IRESv10, and IRESv12. ***B***, Representative confocal images of dendrites expressing each IRES construct, delivered via plasmid lipofection: IRESwt (left), IRESv10 (middle), and IRESv12 (right). Scale bars, 1 µm. ***C***, Distributions of mTq and mVenus intensities in dendritic spines under six conditions (v12, v10, and wt at 1 and 3 µg/ml) before adding A/C heterodimerizer (−5 min). Signals were measured per spine and averaged per dendrite. Gray shading indicates mVenus concentrations that induce unintended baseline changes in spine volume (Fig. 4; 10^0.5^ a.u./µm^3^). Only dendrites with background-subtracted signal intensity ≥0 are shown. Mean ± SEM for each condition are indicated by crosshairs. Linear regression lines are shown for each IRES variant. ***D***, Distributions of mTq intensities in dendritic spines under six conditions (v12, v10, and wt at 1 and 3 µg/ml lipofection). Only dendrites with background-subtracted signal intensity ≥0 are shown. *N* (dendrites/neurons) = 8/6 (wt, 3 µg/ml), 19/11 (v10, 3 µg/ml), 11/5 (v12, 3 µg/ml), 9/7 (wt, 1 µg/ml), 13/9 (v10, 1 µg/ml), and 14/8 (v12, 1 µg/ml). Statistical comparisons were made using the Kruskal–Wallis test (1 µg/ml, *H* = 0.78, *p* = 0.68; 3 µg/ml, *H* = 1.8, *p* = 0.41). ***E***, Expression ratio of mVenus to mTq for the three IRES variants. Signals were measured per spine and averaged per neuron. Statistical comparison between v10 (3 µg/ml) and v10 (1 µg/ml) was performed using the Mann–Whitney test (*U* = 35, *p* = 0.29). ***F***, The concentration range at which SynK constructs linked via each IRES variant induce A/C-dependent spine enlargement without affecting baseline spine volume. Top, Averaged dendritic spine volume before A/C heterodimerizer addition, plotted for each IRES variant across expression levels measured by spine mTq intensity. Data were binned in doubling intervals, with the first bin defined as ≤50 a.u., and the mean ± SEM for each bin is shown. Statistical comparisons were performed using the Mann–Whitney test, comparing each variant to cell-fill–only neurons within each bin, with Bonferroni’s correction. For wt, −50 a.u./µm^3^, *N* (spines) = 36, *U* = 1,469, *p* = 0.13; 50–100 a.u./µm^3^, *N* = 43, *U* = 1,853, *p* = 0.027; 100–200 a.u./µm^3^, *N* = 44, *U* = 2,086, *p* = 8.3 × 10^−4^; 200–400 a.u./µm^3^, *N* = 19, *U* = 954, *p* = 2.5 × 10^−3^. For v10, −50 a.u./µm^3^, *N* = 37, *U* = 1,586, *p* = 0.041; 50–100 a.u./µm^3^, *N* = 69, *U* = 2,775, *p* = 0.094; 100–200 a.u./µm^3^, *N* = 107, *U* = 4,303, *p* = 0.064; 200–400 a.u./µm^3^, *N* = 51, *U* = 1,779, *p* = 0.92; 400–600 a.u./µm^3^, *N* = 12, *U* = 457, *p* = 0.57. For v12, −50 a.u./µm^3^, *N* = 49, *U* = 1,851, *p* = 0.38; 50–100 a.u./µm^3^, *N* = 54, *U* = 1,806, *p* = 0.77; 100–200 a.u./µm^3^, *N* = 79, *U* = 2,844, *p* = 0.65; 200–400 a.u./µm^3^, *N* = 53, *U* = 1,905, *p* = 0.70; 400–600 a.u./µm^3^, *N* = 18, *U* = 646, *p* = 0.80. Middle, The percentage change in dendritic spine volume between 35 and 75 min following A/C heterodimerizer administration, plotted in the same manner. Statistical comparisons were performed using the Mann–Whitney test, comparing each variant to IRESv10 within each bin, with Bonferroni’s correction. For wt, −50 a.u./µm^3^, *U* = 659, *p* = 0.94; 50–100 a.u./µm^3^, *U* = 1,352, *p* = 0.43; 100–200 a.u./µm^3^, *U* = 2,723, *p* = 0.13; 200–400 a.u./µm^3^, *U* = 563, *p* = 0.30. For v12, −50 a.u./µm^3^, *U* = 872, *p* = 0.77; 50–100 a.u./µm^3^*U* = 1,718, *p* = 0.46; 100–200 a.u./µm^3^, *U* = 3,927, *p* = 0.41; 200–400 a.u./µm^3^*U* = 595, *p* = 8.8 × 10^−7^; 400–600 a.u./µm^3^*U* = 33, *p* = 1.6 × 10^−3^. Bottom, For each bin, the mean spine volume before A/C and the mean change in spine volume were calculated. For each IRES variant, bins exhibiting ≥15% increase in spine volume without a significant increase in baseline dendritic spine volume were identified, and the corresponding spine mTq concentration range (minimum to maximum) is indicated by markers. ***G***, Representative confocal image of a dendrite expressing PSDΔ1,2–FRB–mTq–IRESv10–mVenus–FKBP–K7GEF before (−5 min) and after (35 min) application of A/C heterodimerizer. mScarlet signal is shown as a volume marker. Scale bars, 1 µm. ***H, I***, Averaged time courses (***H***) and summary quantification (***I***) of changes in dendritic spine mVenus concentration, quantified as the mVenus-to-mScarlet ratio (top) and spine volume (bottom) from neurons expressing SynK constructs linked via IRESwt, IRESv10, or IRESv12, as well as an IRESv10 GEF-dead mutant (dGEF), following lipofection at 3 µg/ml. mVenus signals for IRESv12 were not plotted due to unreliable detection. Data in ***H*** are shown as the mean ± SEM. Values in ***I*** represent the average between 35 and 115 min after A/C heterodimerizer administration. *N* (dendrites/neurons) = 8/6 (wt), 19/11 (v10), 11/5 (v12), 8/5 (v10 dGEF), and 3/2 (cell-fill only). The median (horizontal line), quartiles (boxes), and range within 1.5 times the interquartile range (whiskers) are denoted. Statistical comparisons were made using the Kruskal–Wallis test (top, *H* = 3.7, *p* = 0.16; bottom, *H* = 16, *p* = 2.5 × 10^−3^) followed by Mann–Whitney test against neurons expressing IRESv10 with Bonferroni’s correction (bottom, wt, *U* = 76, *p* = 1.0; v12, *U* = 164, *p* = 0.011; v10-dGEF, *U* = 126, *p* = 6.5 × 10^−3^, Cell-fill only, U = 23, *p* = 1.3 × 10^−3^). ***J***, Ratio of highly branched spines per dendrite under 3 µg/ml plasmid lipofection. Statistical comparisons were performed using the Kruskal–Wallis test (*H* = 11, *p* = 9.7 × 10^−3^), followed by prespecified Mann–Whitney tests against neurons expressing cell-filling mScarlet alone (without SynK), with Bonferroni’s correction. IRESwt, *U* = 5, *p* = 8.0 × 10^−3^; IRESv10, *U* = 62, *p* = 0.80; IRESv12, *U* = 31, *p* = 0.51. Data for cell-fill only were reproduced from Figure 4*G* for comparison. *N* (dendrites/neurons) = 8/6 (wt), 19/11 (v10), 11/5 (v12), 8/5 (v10 dGEF), and 7/4 (cell-fill only). ***K***, Spine density in neurons expressing SynK linked via IRESv10 following 3 µg/ml lipofection, before and after A/C heterodimerizer administration, quantified per 10 µm of dendrite. *N* (dendrites/neurons) = 19/11 (v10). Wilcoxon signed-rank test (*W* = 12, *p* = 0.71). ***L, M***, Intrinsic spine fluctuation of neurons expressing PSDΔ1,2–FRB–mTq–IRESv10–mVenus–FKBP–K7GEF, before and after A/C heterodimerizer administration, each calculated per 20 min interval. Each plotted point represents the mean (***L***) and the SD (***M***) of spine-head volume changes in 21 pooled spines with similar baseline volumes. Error bars indicate SEM values (***L***) and the 95% confidence intervals of the estimated SD (***M***). Data are fitted by a zero (***L***) and to the 2/3 power of the baseline spine volume (***M***). *p < 0.05; **p < 0.01; n.s., not significant.

We next analyzed structural abnormalities before A/C administration and A/C-induced spine enlargement in SynK constructs linked by each IRES variant while varying the DNA lipofection concentration within a range (1–3 µg/ml) in which PSDΔ1,2–FRB–mTq does not cause morphological abnormalities (Fig. 5). Before A/C heterodimerizer administration, IRESwt, under which mVenus–FKBP–K7GEF expression more readily exceeded the safe range (Fig. 6*C*), exhibited increased spine volume (Fig. 6*F*, top). In contrast, within a similar DNA concentration range, neither mVenus–FKBP–K7GEF expression exceeding this range nor these unintended effects were observed with IRESv10 or IRESv12 (Fig. 6*C*,*F*, top).

Following A/C heterodimerizer administration, mVenus–FKBP–K7GEF translocation to dendritic spines was comparably observed in IRESwt and IRESv10, whereas in IRESv12, the mVenus signal was largely undetectable, precluding reliable assessment of translocation (Fig. 6*G*–*I*). Consistently, spine volume increases were comparable between IRESwt and IRESv10, whereas IRESv12 showed a significantly smaller effect, likely reflecting insufficient mVenus–FKBP–K7GEF expression (Fig. 6*F*, middle, *G–I*).

Across expression titrations for each variant, we defined the effective concentration window (dynamic range) as the fold range between the highest and lowest concentrations that showed no detectable alterations in spine volume prior to A/C heterodimerizer administration while still inducing >15% spine enlargement in an A/C-dependent manner. This dynamic range was largest for IRESv10 (6.0-fold); no such range was observed for IRESwt, and only a narrow range was observed for IRESv12 (1.5-fold). Thus, IRESv10 provides the most balanced expression of the two components, achieving optimal efficacy with minimal baseline morphological perturbation among the variants tested.

In the IRESv10 construct, replacing the GEF domain with a catalytically inactive mutant (dGEF) (Penzes et al., 2001) did not affect A/C heterodimerizer-dependent accumulation in spines (Fig. 6*H*,*I*, top panels) but significantly reduced the extent of spine enlargement (Fig. 6*H*,*I*, bottom panels), suggesting that spine enlargement is dependent on GEF activity. In IRESv10, the proportion of highly branched spines was not significantly increased compared with control neurons lacking SynK expression, whereas it was elevated in IRESwt (Fig. 6*J*). We also examined spine density or intrinsic spine fluctuation before and after A/C heterodimerizer administration, finding no significant unintended changes (Fig. 6*K*–*M*).

We next examined whether spine enlargement induced by the IRESv10-based construct was accompanied by changes in postsynaptic glutamate receptor accumulation. Super-ecliptic pHluorin-tagged GluA2 (SEP–GluA2; Kopec et al., 2006; Penn et al., 2017) was cotransfected with a fluorescent-protein-free version of the IRESv10-based construct. Spine SEP–GluA2 fluorescence increased following A/C heterodimerizer application (Fig. 7*A*–*C*), and the magnitude of this increase correlated significantly with spine enlargement (Fig. 7*D*). These results indicate that structural plasticity induced by the IRESv10-based construct is accompanied by increased surface accumulation of AMPA receptors.

**Figure 7.**
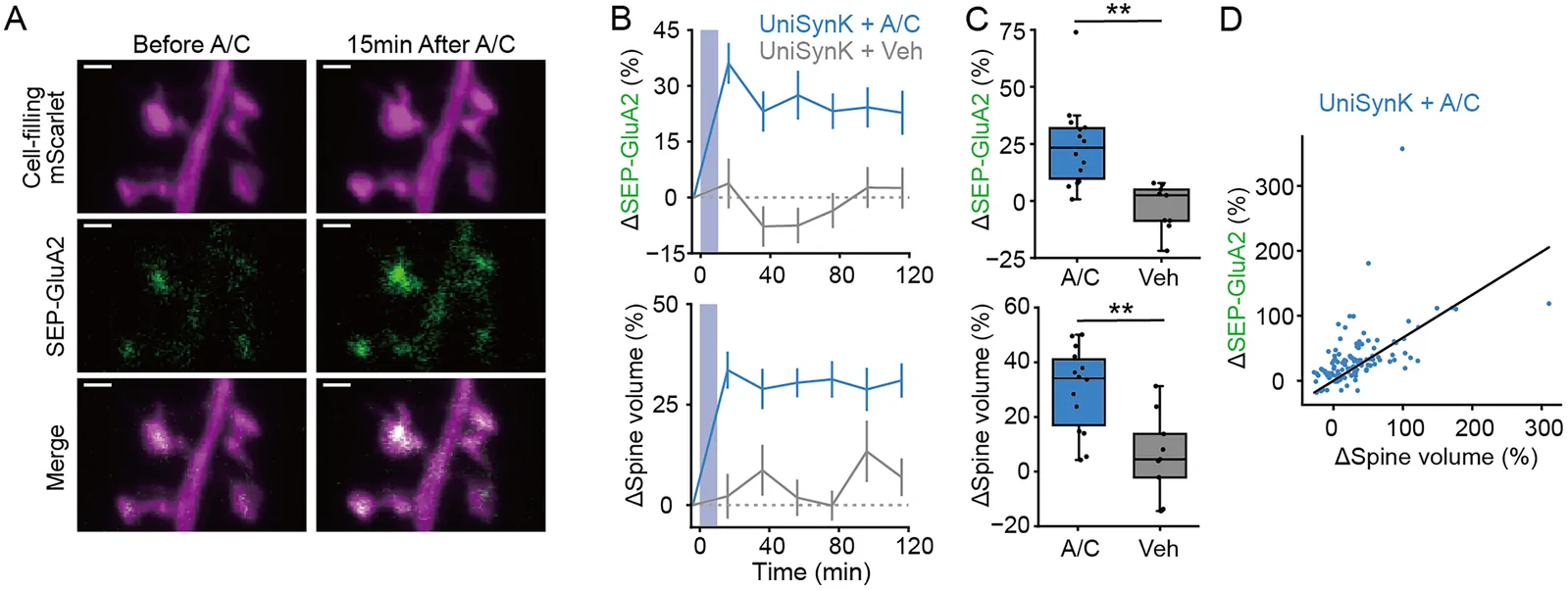
uniSynK-induced spine enlargement is accompanied by increased surface GluA2 accumulation. ***A***, Representative confocal image of a dendrite from a neuron cotransfected with uniSynK (IRESv10; 3 µg/ml) and SEP–GluA2 (2 µg/ml). mScarlet was used as a volume marker. Scale bar, 1 µm. ***B, C***, Averaged time courses (***B***) and summary plots (***C***) of changes in spine SEP–GluA2 signal (top) and spine volume (bottom). Data in ***B*** are shown as the mean ± SEM. Values in ***C*** are averages from 40 and 120 min after administration of A/C heterodimerizer or vehicle. *N* (dendrites/neurons) = 14/6 (uniSynK + A/C) and 9/4 (uniSynK + Veh). The median (horizontal line), quartiles (boxes), and range within 1.5 times the interquartile range (whiskers) are denoted. Mann–Whitney tests: SEP–GluA2, *U* = 119, *p* = 4.7 × 10^−4^; spine volume: *U* = 111, *p* = 2.7 × 10^−3^. ***D***, Relationship between changes in spine volume and SEP–GluA2 signal in the uniSynK + A/C group. Each point represents one spine. Correlation coefficient is 0.52 (*p* = 6.8 × 10^−9^). *p < 0.05; **p < 0.01; n.s., not significant.

### AAV-based validation confirms that IRESv10-linked uniSynK reliably induces spine enlargement with minimal unintended morphological abnormalities

Finally, we termed the IRESv10-based construct uniSynK and compared it with the original split two-component SynK using an AAV-based approach (Fig. 8*A*). To visualize neuronal morphology, mScarlet was sparsely expressed using low levels of Cre recombinase, whereas split SynK or uniSynK was delivered Cre-independently and broadly expressed across infected neurons. When the two components of the original split SynK system were delivered at a 50:1 ratio, the resulting expression ratio between PSDΔ1,2–FRB–mTq and mVenus–FKBP–K7GEF was, on average, comparable to that achieved with IRESv10 in lipofection condition (Fig. 6*E*) but exhibited substantial variability (Fig. 8*B*,*C*). To further examine this variability, cells were categorized into three groups (Groups 1–3) based on their expression ratios (Fig. 8*C*), with Group 2 corresponding to cells within the IRESv10-equivalent range (Fig. 6*E*; 0.21–0.48).

**Figure 8.**
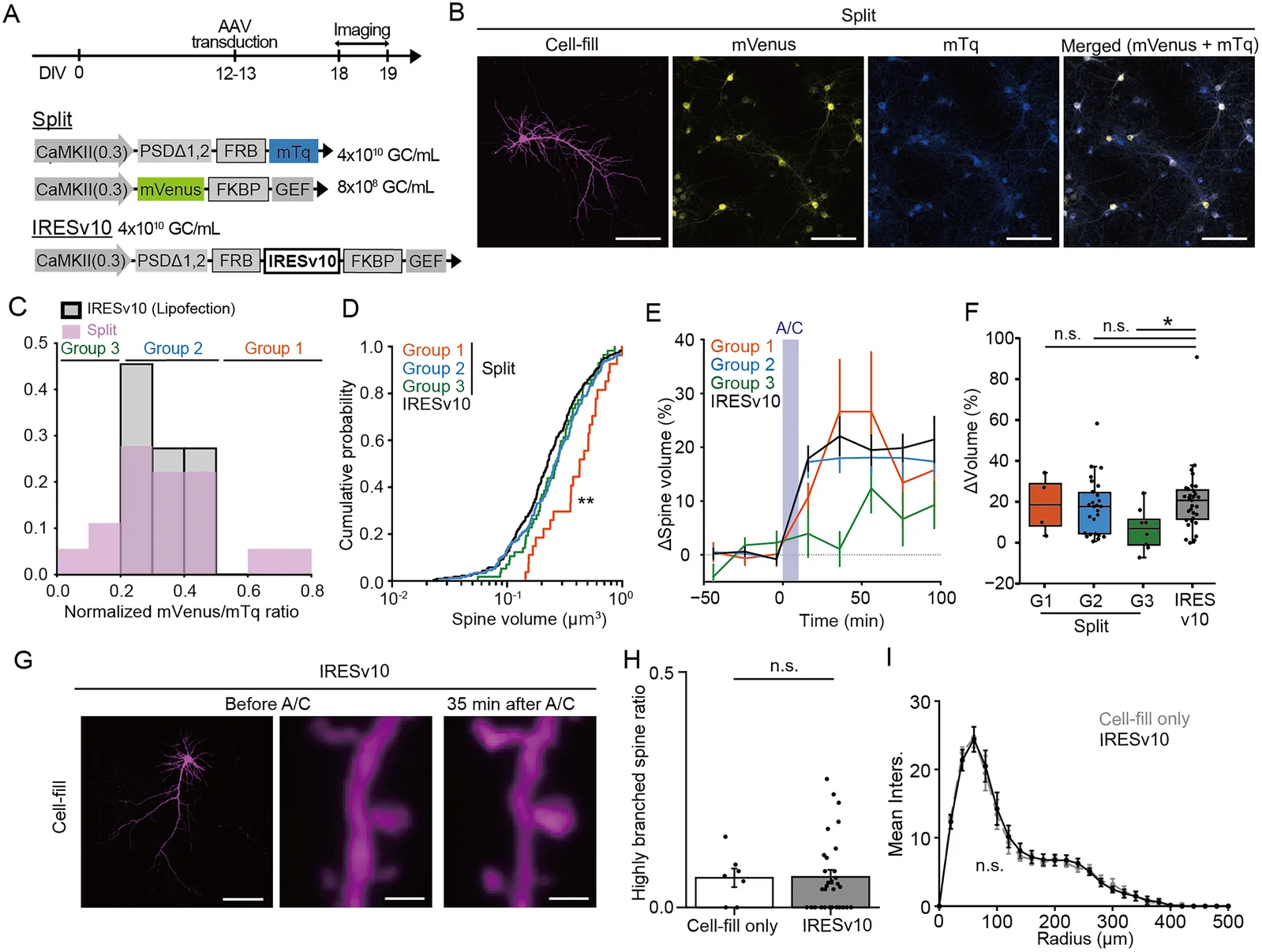
AAV-based validation confirms that IRESv10-linked uniSynK reliably induces spine enlargement with minimal unintended morphological abnormalities under baseline conditions. ***A***, Schematic of uniSynK (single construct linked via IRESv10) and split SynK (two constructs). Constructs were delivered using AAV. ***B***, Representative confocal images of neurons expressing split SynK. For visualization purposes only, higher concentrations were used while maintaining the same delivery ratio (PSDΔ1,2–FRB–mTq, 1 × 10^11^ GC/ml; mVenus–FKBP–K7GEF, 2 × 10^9^ GC/ml). Scale bars, 50 µm. ***C***, Distribution of the mVenus-to-mTq expression ratio in spines for the split construct. Ratios were calculated for individual spines and averaged per cell for plotting. Cells were classified into three groups based on these ratios: within the IRESv10 range defined under 3 µg/ml plasmid lipofection (including both mTq and mVenus; Fig. 6*E*; group 2), above this range (group 1), and below this range (group 3). ***D***, Cumulative distributions of dendritic spine volumes under four conditions: uniSynK and split SynK Groups 1–3. *N* (spines/dendrites/neurons) = 227/29/16 (IRESv10), 27/4/2 (Group 1), 175/26/12 (Group 2), and 57/8/4 (Group 3). Statistical comparisons were performed using the Kolmogorov–Smirnov (K–S) test against the uniSynK condition with Bonferroni’s correction. Group 1, *D* = 0.44, *p* = 9.6 × 10^−5^; Group 2, *D* = 0.12, *p* = 0.097; Group 3, *D* = 0.13, *p* = 0.39. ***E, F***, Averaged time courses (***E***) and summary quantification (***F***) of changes in spine volume from neurons expressing uniSynK and split SynK Groups 1–3. Data in ***E*** are shown as the mean ± SEM. Values in ***F*** represent the average between 15 and 95 min after A/C heterodimerizer administration. *N* (dendrites/neurons) = 31/16 (uniSynK, IRESv10), 4/2 (split SynK Group 1), 26/12 (split SynK Group 2), and 8/4 (split SynK Group 3). The median (horizontal line), quartiles (boxes), and range within 1.5 times the interquartile range (whiskers) are denoted. Statistical comparisons were made using the Mann–Whitney test against the uniSynK condition with Bonferroni’s correction. Group 1, *U* = 61, *p* = 0.98; Group 2, *U* = 443, *p* = 0.52; Group 3, *U* = 198, *p* = 8.6 × 10^−3^. ***G***, Representative confocal images of a neuron (left) and a dendrite expressing uniSynK before (−5 min, middle) and after (35 min, right) application of A/C heterodimerizer. mScarlet signal is shown as a volume marker. Scale bars, 100 µm (left), 1 µm (middle, right). ***H***, Fraction of highly branched spines per dendrite under AAV transduction. Mann–Whitney test: *U* *=* 124, *p* = 0.58, *N* = 7 (Cell-fill only) and 31 (uniSynK, IRESv10) dendrites. ***I***, Sholl analysis showing the number of dendrite intersections as a function of the distance from the soma. Data are presented as mean ± SEM. Data for cell-fill only were reproduced from Figure 4*I* for comparison. Two-way mixed ANOVA: Group × Radius interaction, *F* = 0.14, *p* = 1.0. *N* = 12 (Cell-fill only), 8 (uniSynK). *p < 0.05; **p < 0.01; n.s., not significant.

In Group 1, where mVenus–FKBP–K7GEF expression was relatively high, increased spine volume was evident prior to A/C application (Fig. 8*D*), consistent with the IRESwt condition (Fig. 6*F*), suggesting baseline overactivation. In contrast, in Group 3, where mVenus–FKBP–K7GEF expression was relatively low, no significant changes in baseline spine volume were observed (Fig. 8*D*), but A/C-induced spine enlargement was attenuated (Fig. 8*E*,*F*), consistent with the IRESv12 condition (Fig. 6*F*,*H*). In Group 2, significant spine enlargement was induced without detectable unintended effects in baseline conditions (Fig. 8*D*–*F*). Together, these results further confirm that precise control of the expression ratio between the two components is critical for achieving safe and effective induction of spine enlargement.

In contrast, a single AAV construct linking the two components via IRESv10 (uniSynK; 4.6 kb)—lacking mVenus and mTq due to packaging constraints—produced significantly fewer morphological abnormalities under baseline conditions than split Group 1 and levels comparable to Groups 2 and 3 (Fig. 8*D*). Moreover, uniSynK exhibited A/C-dependent spine enlargement comparable to Groups 1 and 2 and greater than Group 3 (Fig. 8*E*,*F*), demonstrating stable and safe induction of spine enlargement. The proportion of highly branched spines and dendritic arbor complexity were not significantly altered by uniSynK expression (Fig. 8*G*–*I*). Among all dendrites analyzed, 71% of uniSynK-expressing dendrites met both of the following criteria: baseline spine volume before A/C addition was within ±2 SD of the control distribution, and mean spine volume increased by >10% following A/C addition. In contrast, only 41% of dendrites expressing split SynK met both criteria. These results indicate that the combined vector improves the reliability of inducible spine enlargement while minimizing unintended baseline morphological abnormalities, likely by constraining variability in the relative expression levels of the two components.

## Discussion

In this work, we present uniSynK, a new tool to induce dendritic spine enlargement by integrating the two separate components of the original SynK (Sawada et al., 2024) into a single construct. When multiple AAVs are used, it is inherently challenging to achieve coordinated expression of all components within their respective optimal ranges across cells (Grieger et al., 2006; Tian et al., 2009; Chen et al., 2013; Maturana et al., 2023). In the original SynK system, this limitation was particularly pronounced for the FKBP–K7GEF component, which required low expression levels to function properly; accordingly, its AAV titer had to be reduced, making it difficult to achieve sufficient transduction across a large population of neurons.

To address this, we first quantitatively defined the expression-level thresholds at which each component produced unintended effects (Figs. 4,5). We then incorporated IRES variants with graded translational efficiencies (Koh et al., 2013) to fine-tune the expression ratio of the two components, enabling stable and robust expression within the optimal functional range (Fig. 6). Notably, we found that these IRES variants provided stable relative expression levels in neurons as well (Fig. 6*E*). The resulting IRESv10-based construct, uniSynK, enabled A/C-dependent spine enlargement over a relatively broad concentration range without evident off-target effects (Figs. 6*E*, 7).

Precise control of relative protein expression is a central requirement when designing coexpression systems. One conventional approach is to combine promoters of different strengths; however, placing multiple promoters in close proximity within a single vector can cause transcriptional interference and thereby compromise the predictability of transgene expression (Eszterhas et al., 2002; Schlatter et al., 2005). Splicing-based strategies can also be used to modulate expression ratios between two genes (Fallot et al., 2009; Aebischer-Gumy et al., 2024). One limitation is that splice-site usage depends on both local sequence context, including cis-acting splicing enhancers and silencers, and the cellular repertoire of splicing regulatory factors, limiting the general applicability of this approach for maintaining stable expression ratios across different transgene pairs (Renaud-Gabardos et al., 2015).

The 2A peptides and IRESs are widely used alternatives for coexpressing multiple genes from a single mRNA. Because of their short length, 2A peptides are particularly useful under AAV packaging constraints (Ryan et al., 1991; de Felipe et al., 2006), and their utility has also been demonstrated in neurons (Furler et al., 2001). However, incomplete cleavage can generate uncleaved fusion products (de Felipe et al., 2010; Chan et al., 2011; Ho et al., 2013). In the SynK system, such products could potentially cause premature localization of the Kalirin-7 GEF domain to dendritic spines before A/C heterodimerizer addition, increasing the risk of unintended effects. In addition, 2A peptides do not readily enable precise tuning of the relative expression ratio, which becomes problematic when the two components require substantially different expression levels.

We therefore adopted an IRES-based strategy (Renaud-Gabardos et al., 2015), in which the expression ratio can be tuned by selecting IRES variants with different translational efficiencies (Koh et al., 2013). The IRES variants examined here were generated by replacing one or more of the 10th, 11th, and 12th AUG codons within the EMCV IRES with near-cognate initiation codons, such as CUG or GUG. Because translation from the EMCV IRES is initiated predominantly at AUG-11 and, to a lesser extent, at AUG-12, the position and identity of these substitutions likely alter downstream translation efficiency by reducing start-codon recognition, analogous to the lower initiation efficiency of near-cognate codons in cap-dependent translation (Mehdi et al., 1990; Davies and Kaufman, 1992; Kaminski et al., 1994). Although the relatively large size of IRES sequences is a potential drawback, we offset this constraint by successfully shortening the construct. Moreover, although the use of IRES variants for quantitative control of relative protein expression in neurons has not been well established, our results demonstrate that IRES-dependent tuning can be achieved in neurons. These findings support IRES variants as a flexible platform for optimizing multiprotein expression systems, particularly when precise ratiometric control is required.

While quantitatively assessing the side effects of PSDΔ1,2–FRB and FKBP–K7GEF expression, we found that each component displayed distinct modes of unintended effects. PSDΔ1,2 has long been used as a postsynaptic marker (Arnold and Clapham, 1999; Sturgill et al., 2009; Hayashi-Takagi et al., 2015; Fujimoto et al., 2023), yet the consequences of its overexpression had not been systematically quantified. We observed that the spine/shaft ratio was maintained within a certain expression range (Fig. 5*B*–*G*), within which overall cellular architecture remained largely unaffected. However, once PSDΔ1,2 expression exceeded its postsynaptic localization capacity and ectopically leaked into dendritic shafts, catastrophic abnormalities emerged, including highly branched spines and lamellipodia-like structures and an aberrant increase in small spines and filopodia (Fig. 5*H*). In contrast, Kalirin-7 produced more modulatory effects that scaled in a relatively linear fashion with concentration, increasing average spine volume and promoting branched spines and spinules (Fig. 4). Altered expression levels of Kalirin-7 and PSD proteins have been reported in depression, autism, and schizophrenia (Karolewicz et al., 2009; Feyder et al., 2010; Duric et al., 2013; Remmers et al., 2014; Russell et al., 2014; Coley and Gao, 2018; Rodríguez-Palmero et al., 2021), and our quantitative investigation on the graded mode of action may offer new insight into how genetic abnormalities translate into synaptic and, ultimately, behavioral phenotypes.

Although the uniSynK system demonstrated improved performance in cultured neurons, several limitations and opportunities for further refinement remain. First, because of AAV packaging constraints, an additional fluorescent reporter could not be included in the final AAV-uniSynK construct (Fig. 8), precluding direct visualization of expression efficiency at the population level. Nevertheless, the IRES-based architecture ensures stable and reproducible expression ratios between the two components (Fig. 6*E*), which may compensate for this limitation by reducing the need for post hoc selection of cells with appropriate expression levels. Second, the functional effect of uniSynK was not directly assessed electrophysiologically. However, spine enlargement has repeatedly been associated with increased AMPA receptor content and synaptic strength (Matsuzaki et al., 2004; Kopec et al., 2006), including after activation of the original split SynK system (Sawada et al., 2024), and the accompanying increase in SEP–GluA2 observed here is consistent with functional potentiation. Third, although uniSynK was robustly validated in cultured neurons using both lipofection and AAV transduction, the expression ranges established using these delivery methods may not directly translate to in vivo AAV delivery, where absolute expression can vary with AAV serotype, target cell type, and brain region, and excessive expression may produce unintended effects. Empirical optimization will therefore be required for each in vivo application. The single-vector architecture should facilitate this process by ensuring codelivery and constraining the relative expression of the two components, thereby reducing the cotransduction and stoichiometric variability inherent to split-AAV delivery. Further refinements, such as introducing additional structural switching mechanisms into Kalirin-7 or enhancing dimerization-dependent translocation efficiency, may further suppress unintended effects and permit effective activation at lower expression levels.

Despite these remaining limitations, the conceptual framework established here—quantitative definition of expression thresholds and ratiometric control of multicomponent systems—addresses a challenge that extends well beyond the SynK system itself. To date, numerous two-component systems have been developed, ranging from chemically induced translocation tools for diverse cell types (Inoue et al., 2005; Bisaria et al., 2020) to neuron-targeted tools for tagging and manipulation (Idevall-Hagren et al., 2012; Lee et al., 2017; Kim et al., 2020; Hyun et al., 2022). However, their use is often limited by the need to fine-tune the expression levels of each component. Here, we provide a practical solution by leveraging IRES variants to precisely control expression ratios, which could be broadly applied to such systems and facilitate their adoption.

In addition to its broader methodological implications, uniSynK provides a practical means to address long-standing biological questions regarding the causal roles of synaptic plasticity. Synaptic alterations have been implicated in a wide range of conditions, including sleep regulation, psychiatric disorders, and brain tumors. Directly testing the functional consequences of spine and synaptic modifications requires tools that perturb synapses themselves, thereby making synapses causal experimental variables. Compared with optogenetic approaches (Hayashi-Takagi et al., 2015; Goto et al., 2021), synapse-targeted chemogenetic manipulation may offer distinct advantages. Structural manipulation of synapses using light can require extended illumination and high power, increasing the risk of phototoxic effects, whereas ligand-dependent systems enable sustained modulation following a single administration without continuous intervention. Moreover, systemic delivery of an otherwise inert chemical dimerizer allows effective manipulation across large brain regions—or even the entire brain—without the need for invasive optical access. When combined with cell type–specific enhancers (Challis et al., 2022; Ben-Simon et al., 2025) and synapse-tagging strategies (Hayashi-Takagi et al., 2015; Gobbo et al., 2017) that selectively label defined synaptic populations, uniSynK could be further extended to manipulate specific synapse subsets, providing a versatile framework for dissecting synapse-specific functions across intact neural circuits.

In summary, uniSynK establishes a generalizable strategy for precise and balanced control of multicomponent systems, enabling robust synaptic actuation.

## Synthesis

Reviewing Editor: Rosemary Bagot, McGill University Decisions are customarily a result of the Reviewing Editor and the peer reviewers coming together and discussing their recommendations until a consensus is reached. When revisions are invited, a fact-based synthesis statement explaining their decision and outlining what is needed to prepare a revision will be listed below. The following reviewer(s) agreed to reveal their identity: NONE.

The authors developed a single-vector version of SYNCit-K (uniSynK), a tool that enables spine enlargement and synaptic potentiation in individual neurons. To minimize off-target effects, the authors first determined the maximal expression levels of each component that did not induce detectable side effects under basal conditions. They then redesigned SynK as a single-vector construct by linking the two components with an internal ribosome entry site (IRES). Furthermore, they optimized the FKBP/FRB expression ratio, allowing both components to be expressed near their maximal effective levels without detectable adverse effects. Finally, they demonstrated that uniSynK exhibits fewer side effects than the original two-vector version while retaining its ability to induce spine enlargement in cultured neurons.

This study addresses a common limitation of AAV-based methodologies and provides an improved tool that will be useful for linking synaptic plasticity to behavior. Although the current study is limited to cultured neurons, the data are of high quality, and the overall manuscript is close to being suitable for publication. I have only a few points that the authors should address before publication.

- Major points

1. Functional assessment of synaptic transmission

To quantitatively determine the appropriate expression levels of the two SynK components, the authors carefully examined dose-dependent effects on spine enlargement in Figures 4-7. These experiments provide valuable information for defining the optimal ratio between the two constructs. However, throughout the manuscript, the authors assess only structural changes in dendritic spines. Although numerous previous studies have demonstrated a strong relationship between spine structure and synaptic function, it would have strengthened the study to evaluate basal synaptic transmission, such as mEPSCs or sEPSCs, using electrophysiological recordings. The authors should discuss potential effects of uniSynK on synaptic transmission in the Discussion section.

2. Limitations associated with gene delivery methods

The authors systematically compare DNA lipofection and AAV-mediated gene delivery systems in a dose-dependent manner. However, transfection efficiency following lipofection is known to vary substantially across cell types and experimental conditions. Similarly, in AAV-mediated gene delivery, viral serotype can strongly influence transduction efficiency across different cell types and brain regions. These factors may ultimately affect transgene expression levels in vivo. Since the authors aim to apply uniSynK in living animals, it would be beneficial to discuss the limitations arising from differences in gene delivery methods and how they may affect in vivo implementation.

3. Mechanistic basis of IRES-dependent expression differences

The authors carefully compared several IRES variants in Figure 6. However, it remains unclear why these variants produce different levels of bicistronic expression, raising concerns about potential overexpression beyond the optimal range. Although elucidating the underlying mechanism is likely beyond the scope of the current study, it would be helpful for the authors to discuss possible explanations for these differences and their implications for controlling expression levels in the Discussion section.

4. Quantification of aberrant spine morphology in single-chain uniSynK

In Figure 7, the authors present data from a single concentration (4 × 10^10 GC/mL) of the single-chain uniSynK construct (Fig. 7D-F). Although IRESv10 is expected to produce lower expression of the downstream component than the other variants, this viral concentration appears sufficiently high to induce highly branched spines in neurons expressing the FKBP-GRF construct alone, based on the data shown in Figures 4F and 4G. Because the exact expression levels of the individual components in the single-chain construct are unknown, it would be informative to quantify the proportion of highly branched spines in neurons expressing uniSynK, similar to the analyses presented in Figures 4F and 4G.

5. Assessment of dendritic branching

The authors thoroughly evaluated spine-related side effects resulting from expression of the two SynK components. However, no analysis of dendritic branching is provided. Since the previous studies have reported abnormal dendritic branching in neurons overexpressing Kalirin-7 (Ma, X.-M. et al., J. Neurosci., 2008), it would be valuable to assess dendritic branching in neurons expressing each SynK component as well as uniSynK.

- Minor Points

1. On lines 228-229, the term "safe expression range" sounds somewhat vague. It may be clearer to use terms such as "optimal expression level" or "effective expression range."

2. Panel E appears to be missing from Figure 6.

3. In Figures 4B and 4D, it would be helpful to show a comparable number of representative images for both DNA lipofection and AAV-mediated delivery (e.g., three representative images from each condition).
